# Surface measurement of total solar and ultraviolet irradiance and ancillary meteorological data at the South-West Indian Ocean Solar network (IOS-net) stations

**DOI:** 10.1016/j.dib.2021.107136

**Published:** 2021-05-15

**Authors:** Béatrice Morel, Patrick Jeanty, Mathieu Delsaut, Nicolas Hassambay, Alexandre Graillet, Jean-Pierre Chabriat

**Affiliations:** LE^2^P-ENERGY lab, Université de La Réunion, 15 avenue René Cassin, CS 92003, Saint-Denis Cedex 9, La Réunion Island 97744, France

**Keywords:** Surface (total, UV) solar irradiance, Pyranometer, UV radiometer, Meteorological information, Weather transmitter, South-West Indian Ocean

## Abstract

The observational data described in this article are collected at several locations in the South-West Indian Ocean (SWIO). Platforms equipped with radiometers and a weather transmitter, and located over Comoros, Madagascar, Mauritius, La Réunion and Seychelles islands, are used to measure incident global and diffuse shortwave radiation and incident global UV *A* + *B*-band radiation along with air temperature, relative humidity, wind speed and direction, air pressure and rainfall amount with a sampling frequency of 0.1 Hz. The data are stored as 1-min averages and automatically transmitted to the LE^2^P-ENERGY lab at the University of La Réunion. The dataset is hosted on the website: https://galilee.univ-reunion.fr, and is uploaded to Zenodo repository. Such a dataset will help in providing information related to solar energy forecasting and assessment for solar energy implementation at a regional and national level in the SWIO.

## Specifications Table

**Table:** 

Subject	Renewable Energy, Sustainability and the Environment
Specific subject area	Solar-energy forecasting and assessment
Type of data	Tables
How data were acquired	All variables are measured *in-situ* on instrumented structures at ground level (< 2-m high). Global and diffuse solar and global UV *A* + *B*-band irradiances (in W/m^2^) are measured using a Delta-T Devices SPN1 pyranometer and a Kipp&Zonen SUV5 UV radiometer, respectively. Air pressure (in hPa), air temperature (in °C), relative humidity (in %), rainfall amount (in mm), wind speed (in m/s) and direction (in °) are obtained with a Vaisala WXT530 Series weather transmitter located at a distance of ~1.5 m from the radiation sensors.
Data format	Raw (NetCDF and csv)
Parameters for data collection	Data are collected continuously at different sites over the islands of the South-West Indian Ocean since the installation of the sensors at the sites of study. Data publicly available include ten sites: three on Mauritius (including Rodrigues island), two on Comoros, Madagascar and Seychelles, and one on La Réunion.
Description of data collection	Solar and weather instruments are connected to a Campbell Scientific CR1000X datalogger for data collection. Data transmission is ensured via TCP/IP protocol or GPRS network. Instantaneous measurements are made for each variable every 10 s, but only the average over one minute is stored on the server.
Data source location	Data are collected at ten stations of the Indian Ocean Solar network (IOS-net): Comoros: • Hahaya, Grande Comore (11.539°S, 43.278°E)-Dec. 2019-*pres.* • Ouani, Anjouan (12.133°S, 44.429°E)-Dec. 2019*-pres.* La Réunion: • La Plaine des Palmistes (21.137°S, 55.624°E)-Dec. 2018-*pres.* Madagascar: • Antananarivo (18.898°S, 47.546°E)-Nov. 2019-*pres.* • Antsiranana (12.349°S, 49.293°E)-Nov. 2019-*pres.* Mauritius: • Bras d'Eau, Mauritius (20.139°S, 57.726°E)-Nov. 2015-*pres.* • Plaine Corail, Rodrigues (19.758°S, 63.371°E)-May 2017-*pres.* • Vacoas, Mauritius (20.297°S, 57.497°E)-Oct. 2019-*pres.* Seychelles: • Amitie, Praslin (4.321°S, 55.693°E)-Nov. 2019-*pres.* • Anse Boileau, Mahé (4.711°S, 55.485°E)-Nov. 2019-*pres.*
Data accessibility	Data which are accessible via the University of La Réunion (https://galilee.univ-reunion.fr), have been uploaded on Zenodo repository (https://zenodo.org) with data DOI's assignment: • Hahaya – http://doi.org/10.5281/zenodo.4408671 • Ouani – http://doi.org/10.5281/zenodo.4408669 • La Plaine des Palmistes – http://doi.org/10.5281/zenodo.4408677 • Antananarivo – http://doi.org/10.5281/zenodo.4408673 • Antsiranana – http://doi.org/10.5281/zenodo.4408675 • Bras d'Eau – http://doi.org/10.5281/zenodo.4408660 • Plaine Corail, Rodrigues – http://doi.org/10.5281/zenodo.4408664 • Vacoas – http://doi.org/10.5281/zenodo.4408662 • Amitie – http://doi.org/10.5281/zenodo.4408667 • Anse Boileau – http://doi.org/10.5281/zenodo.4408665

## Value of the Data

• Accurate knowledge of the quantity and spatiotemporal variability of the solar resource at given locations needs to be addressed to enable integration of high levels of solar power (photovoltaic or PV systems) into the national utility grids. This is especially the case for the South-West Indian Ocean (SWIO) Islands (Comoros, La Réunion, Madagascar, Mauritius and Seychelles) where accurate ground-based Surface Solar Radiation (SSR) measurements with high temporal resolution (~1 min) remain sporadic. Given the objective of these islands to become self-sufficient for their electricity with renewable energies, solar-energy assessment and forecasting are essential for solar energy project policy and planning.
• Global horizontal irradiance (GHI) and diffuse horizontal irradiance (DHI) are helpful for translation of the GHI to in-plane irradiance as required by the majority of PV systems.
• Single or combined use of the data allows for estimation of PV output power – either indirectly by means of a physical model that converts the measured global horizontal irradiance into electricity, or directly by using the meteorological data (air pressure, temperature, relative humidity, wind, rain) as input variables to machine learning algorithms. Data contained herein also allows for analysis of PV module performance losses due to temperature, relative humidity and UV irradiation under different climatic conditions.
• Data contained herein allows for comparison of ground-based measurements of solar irradiance on the SWIO Islands against values derived from satellite observations or simulated with regional climate models.

## 1 Data Description

**Fig. 1:**
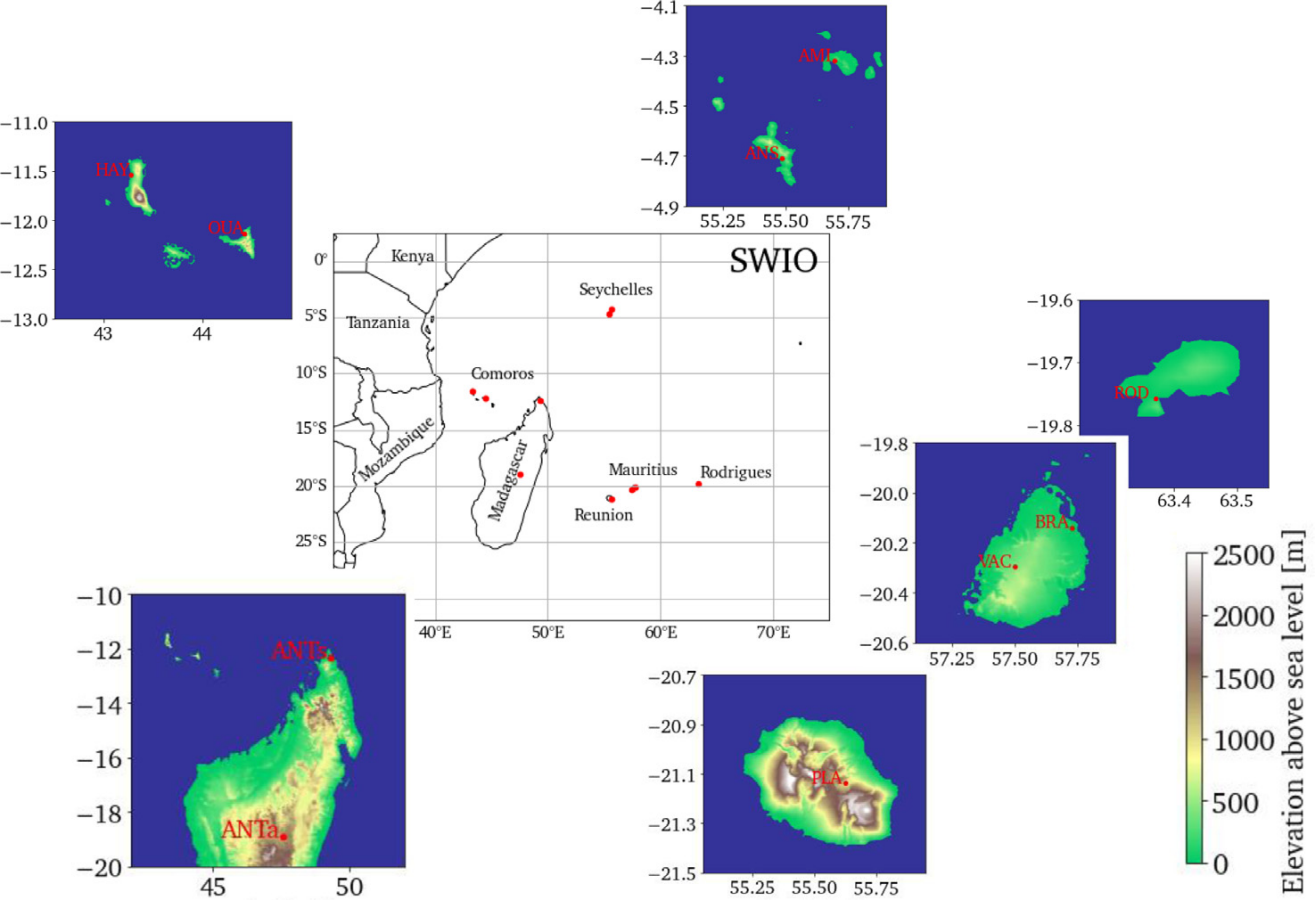
Map of the South West Indian Ocean region. Red dots indicate the 10 radiometric stations of the Energy-Lab network whose data are presented in the present paper. The locations are shown in more detail in the inset maps of topography from SRTM15+ digital elevation model ([2]).

**Fig. 2:**
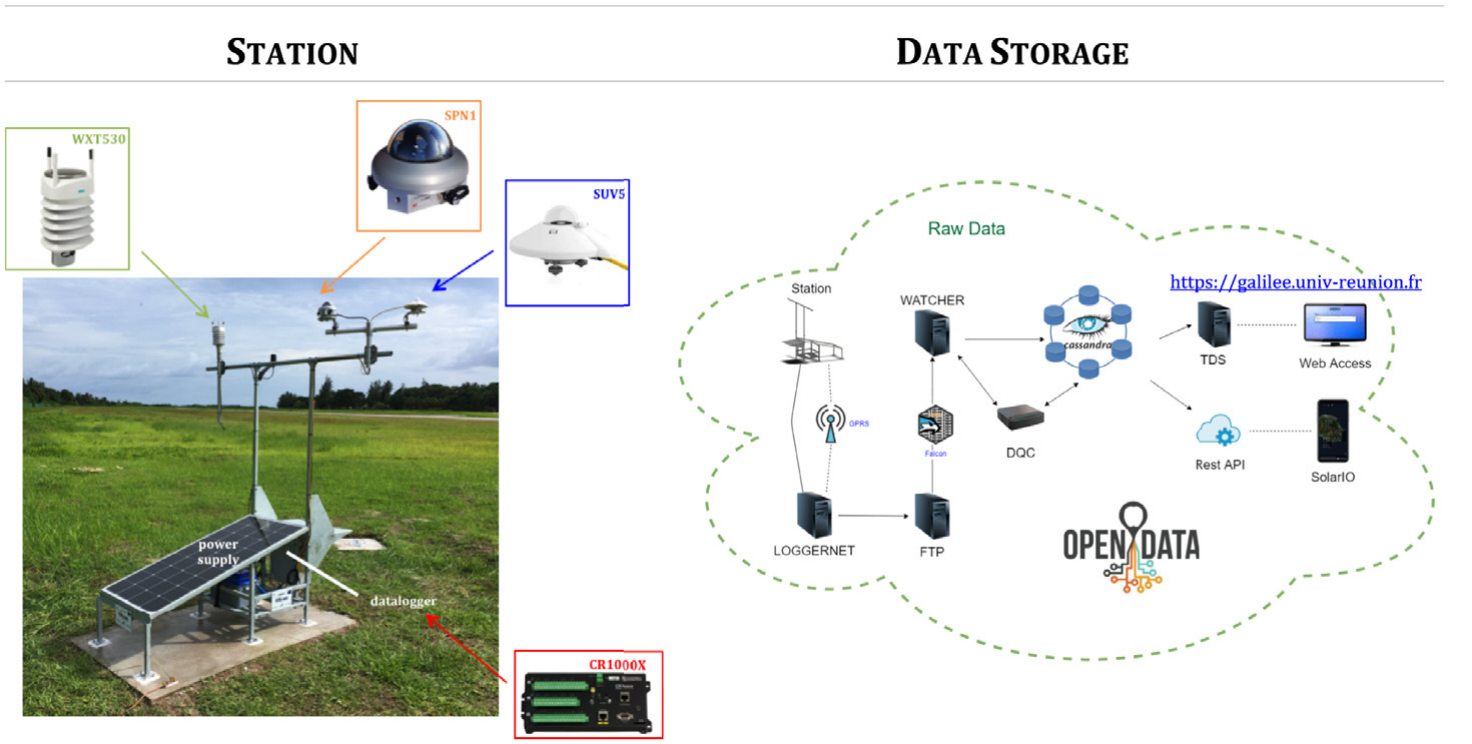
Instrumentation of the stations and architecture of the database of the IOS-net network.

The data are collected at the radiometric stations of the network developed by the LE^2^P-ENERGY lab in the SWIO since the early 2010s ([1]). The dataset readily available with this article includes observed meteorological and SSR data from 10 sites on the insular territories covered by the Indian Ocean Commission: Comoros, La Réunion, Madagascar, Mauritius and Seychelles (Fig. 1). The data are gathered since the end of 2019 at all these sites except Bras d'Eau in Mauritius (2015), Plaine Corail in Rodrigues (2017), and La Plaine des Palmistes in La Réunion (2018; see also Table 1) using multi-instrumented sensor platforms as displayed in Fig. 2. The SSR data are presented in terms of global and diffuse shortwave and global UV-*A* + *B* irradiances, and are expressed in W/m^2^. The meteorological data include air temperature (in °C), air relative humidity (in %), wind speed (in m/s) and direction (in °), air pressure (in hPa) and rainfall amount (in mm). The data, with sampling frequency of 0.1 Hz, are stored as 1-min averages by a datalogger. The 1-min average data are hosted in both NetCDF and csv formats on a THREDDS Data Server (TDS) created in the framework of the Indian Ocean Solar Network (IOS-net) project funded by the European INTERREG V Indian Ocean (2014–2020) and the Indian Ocean Commission Energies programmes (www.commissionoceanindien.org), and currently located at: https://galilee.univ-reunion.fr. Information about the dataset (including all abbreviations appearing in the data files) is given in the NetCDF dataset which includes comprehensive CF-convention metadata. Ultimately, the data collected by the LE^2^P-ENERGY lab at all the sites of its network will be made public through this TDS. In addition, the NetCDF dataset has also been uploaded on the international data repository Zenodo: https://zenodo.org. While the dataset on Zenodo is updated on a yearly basis (that is on the 1^st^ of January of each year), the TDS offers the most up to date version of the dataset and includes data recording up to the day before.

**Table 1:** Location of the 10 IOS-net stations described in the present paper.

Site	Longitude (°E)	Latitude (°S)	Height (m)	Measurement period
**Comoros**				
Hahaya, Grande Comore (HAH)	43.278	11.539	33	5 Dec 2019-*present*
Ouani, Anjouan (OUA)	44.429	12.133	20	6 Dec 2019-*present*
**La Réunion**				
La Plaine des Palmistes (PLA)	55.624	21.137	1054	12 Dec 2018-*present*
**Madagascar**				
Antananarivo (ANTa)	47.546	18.898	1306	19 Nov 2019-*present*
Antsiranana (ANTs)	49.293	12.349	108	21 Nov 2019-*present*
**Mauritius**				
Bras d'Eau, Mauritius island (BRA)	57.726	20.139	24	5 Nov 2015-*present*
Plaine Corail, Rodrigues island (ROD)	63.371	19.758	36	9 May 2017- *present*
Vacoas, Mauritius island (VAC)	57.497	20.297	430	29 Oct 2019-*present*
**Seychelles**				
Anse Boileau, Mahe (ANS)	55.485	4.711	3	18 Nov 2019-*present*
Amitie, Praslin (AMI)	55.693	4.321	3	20 Nov 2019-*present*

## 2 Experimental Design, Materials, and Methods

The data are obtained from 10 sites over the SWIO (Fig. 1 and Table 1). The special locations of the measuring sites on the islands of the SWIO make those sites challenging places for solar energy studies and applications. Each measurement station has roughly the same design including autonomous power supply based on renewable (solar) energy. The dataset was obtained using (Table 2):

– a Delta-T Devices SPN1 pyranometer which simultaneously measures GHI and DHI (in W/m^2^),
– a Vaisala WXT530 Series weather transmitter located at a distance of 1.5 m from the pyranometer, which measures air pressure (in hPa), air temperature (in °C), relative humidity (in %), wind speed (in m/s) and direction (in °), and rainfall (in mm),

mounted on a structure at ground level (2-m high; Fig. 2). Except for Bras d'Eau (Mauritius), Plaine Corail (Rodrigues) and La Plaine des Palmistes (La Réunion), the measurement stations also include a Kipp&Zonen SUV5 UV radiometer which measures the global UV *A* + *B*-band irradiance (in W/m^2^). All sensors are connected to a Campbell Scientific CR1000X datalogger for data collection. Data transmission to the server is ensured via TCP/IP protocol or GPRS network. Data presented herein are raw data. Thus, we highly recommend that all users perform their own quality checks of the data before using them (e.g., [3]).

**Table 2:** Equipment installed at the IOS-net stations.

Variable (unit)	Sensor *Manufacturer*	Comment
Incident shortwave global and diffuse irradiance (W/m^2^)	SPN1 *Delta-T Devices*	at all stations
Incident global ultraviolet UV-*A* + *B* irradiance (W/m^2^)	SUV5 *Kipp and Zonen*	at all stations except Bras d'Eau (BRA), Plaine Corail (ROD) and La Plaine des Palmistes (PLA)
Air temperature (°C)	WXT530 series	multi-parameter weather transmitter, at all stations
Air pressure (hPa)	*Vaïsala*	
Rainfall amount (mm)		
Relative humidity (%)		
Wind speed (m/s^−1^) and direction (°)		

## CRediT Author Statement

**Béatrice Morel:** Conceptualization, Writing – original draft; **Patrick Jeanty:** Investigation, Resources; **Mathieu Delsaut:** Data curation, Visualization; **Nicolas Hassambay:** Investigation, Resources; **Alexandre Graillet:** Resources, Data curation, Visualization; **Jean-Pierre Chabriat:** Supervision, Project administration, Funding acquisition.

## Declaration of Competing Interest

The authors declare that they have no known competing financial interests or personal relationships, which have, or could be perceived to have, influenced the work reported in this article.

## References

[1] M. Delsaut, P. Jeanty, B. Morel, D. Gay, “J'veux du soleil”: towards a decade of solar irradiation data (La Réunion Island, SW Indian Ocean), In: Lemaire V., Malinowski S., Bagnall A., Guyet Th., Tavenard R., Ifrim G. (eds.) Advanced Analytics and Learning on Temporal Data –5th ECML PKDD Workshop, AALTD 2020, Ghent, Belgium, September 18, 2020, Revised Selected Papers. Springer, Berlin, Heidelberg, 2020.

[2] B. Tozer, D.T. Sandwell, W.H.F. Smith, C. Olson, J.R. Beale, P. Wessel, Global bathymetry and topography at 15′: SRTM15+, earth and space science 6 (2019), doi:10.1029/2019EA000658.

[3] Yang D., Yagli G.M., Quan H. (2018). Quality control for solar irradiance data, 2018 IEEE innovative smart grid technologies-Asia (ISGT Asia). Singapore.

